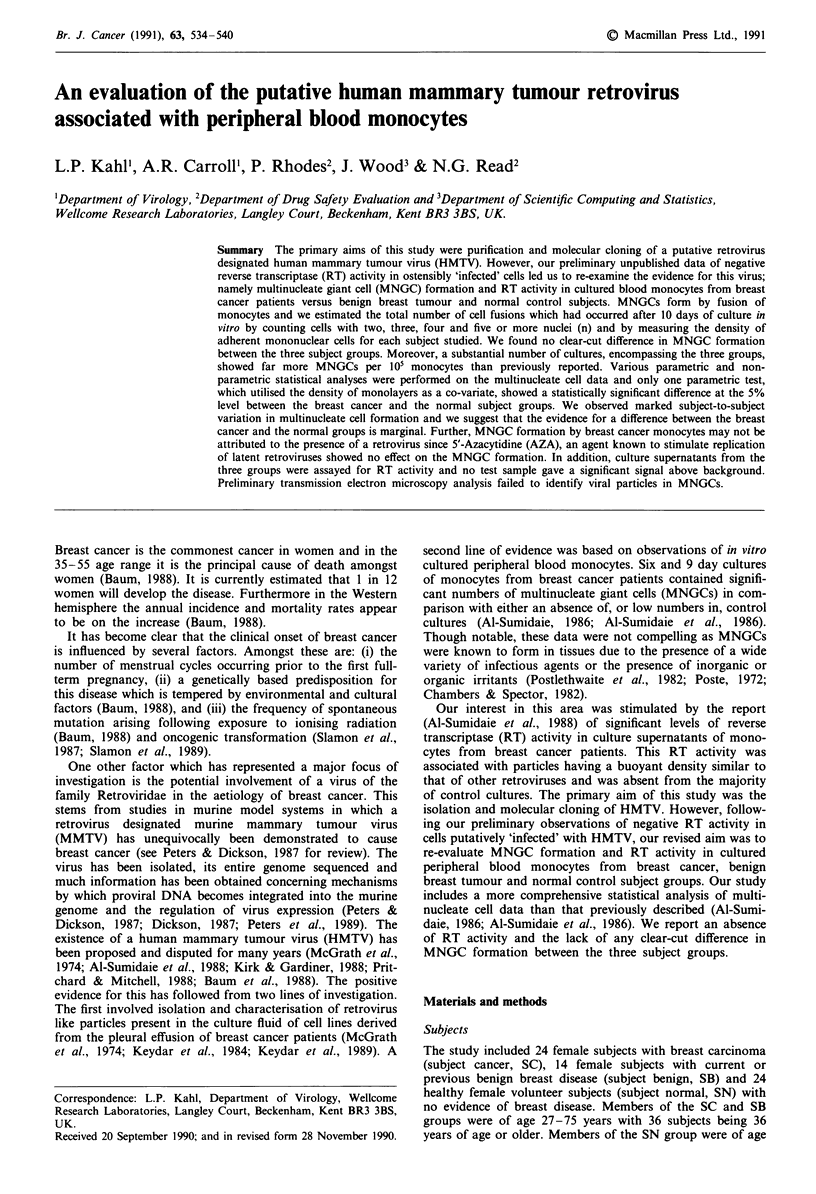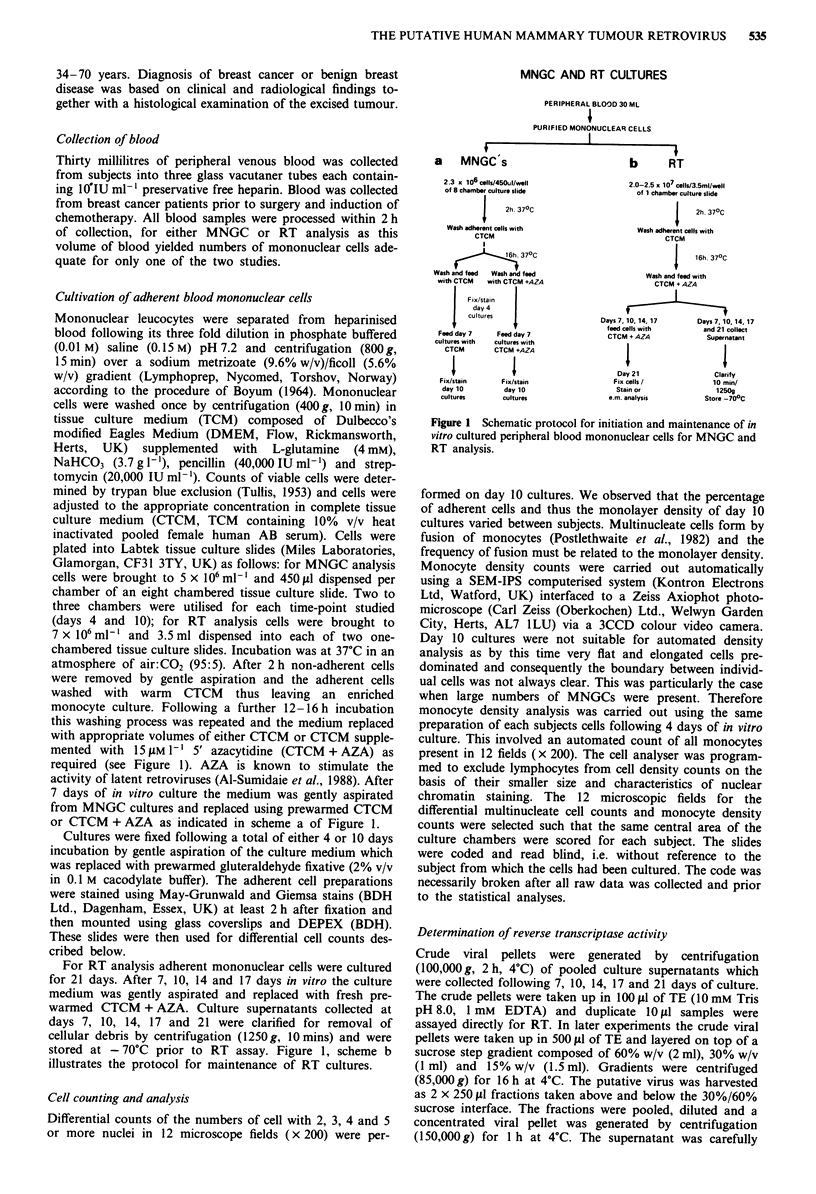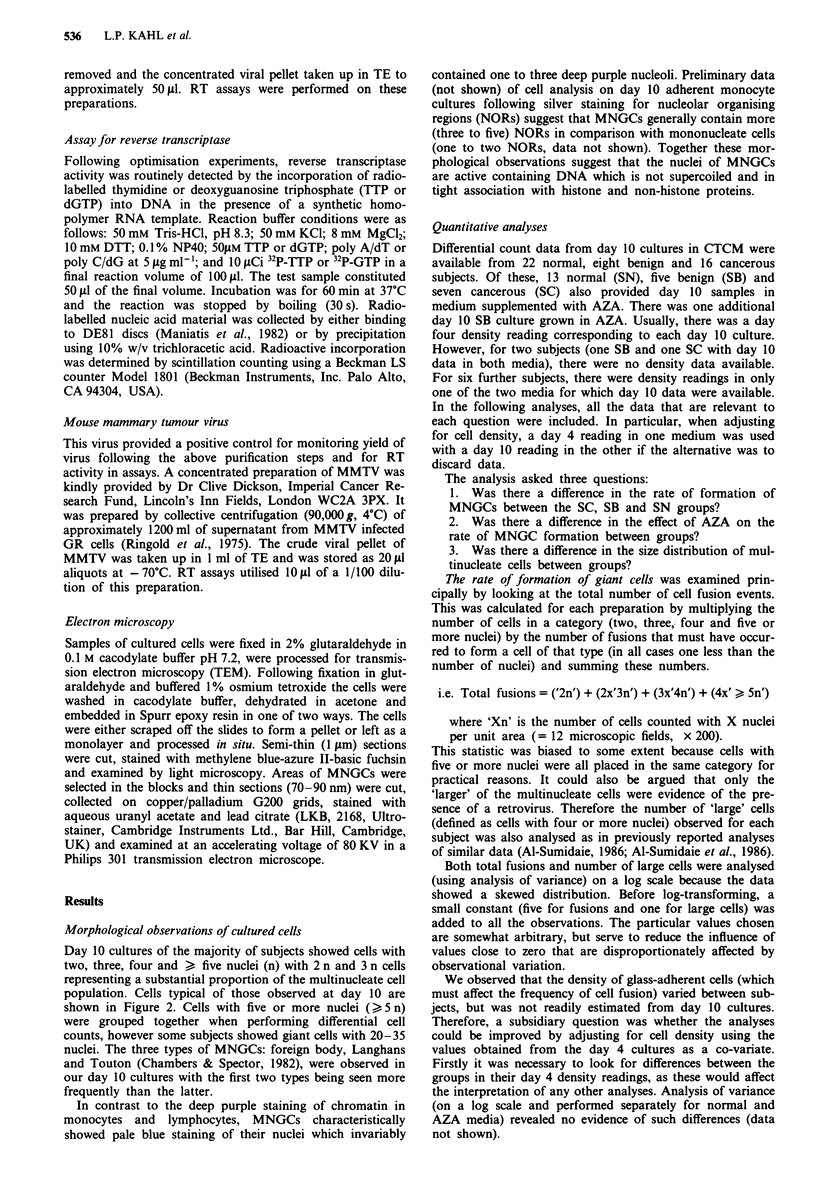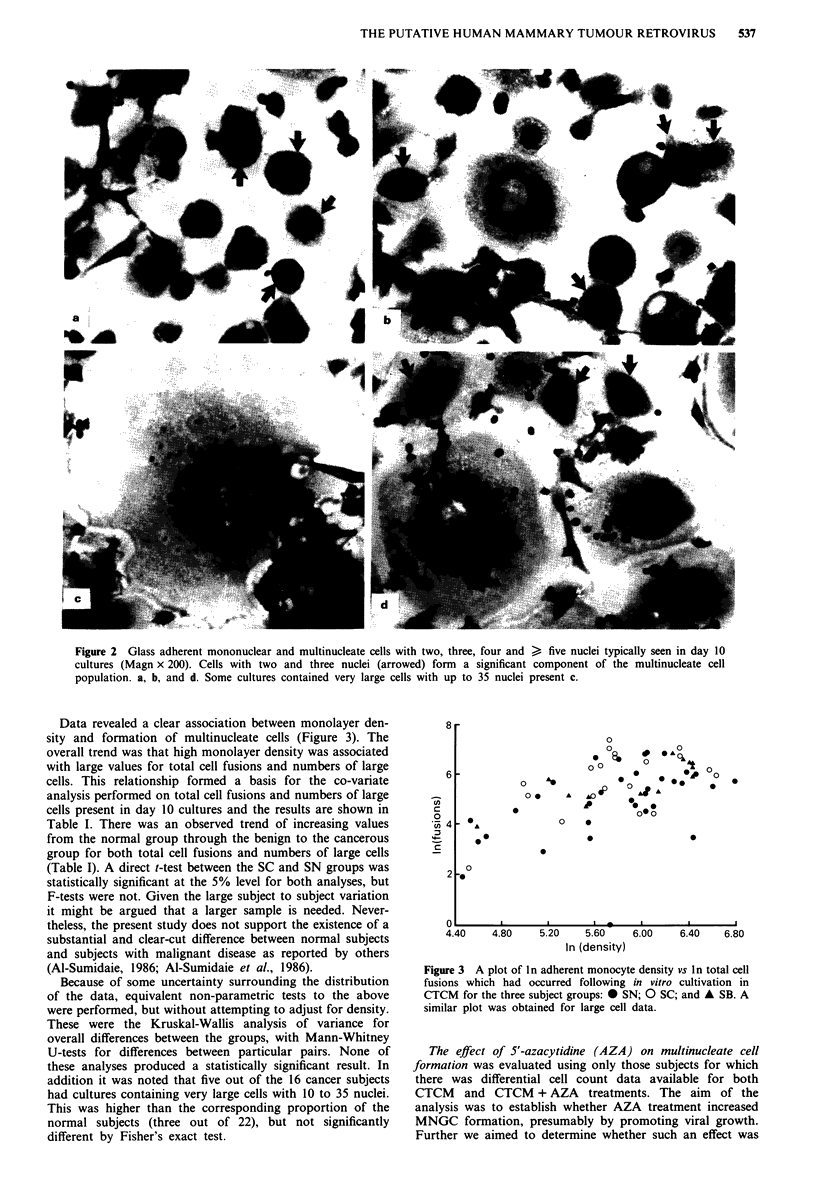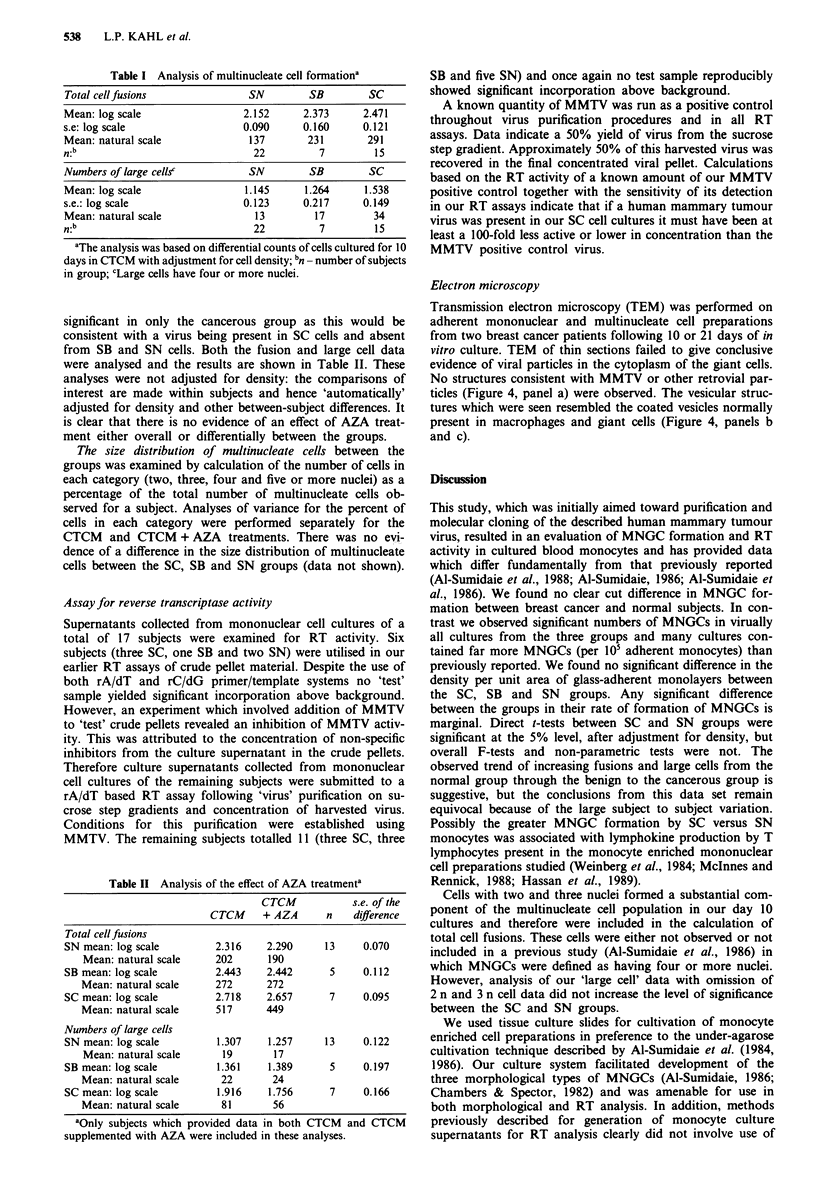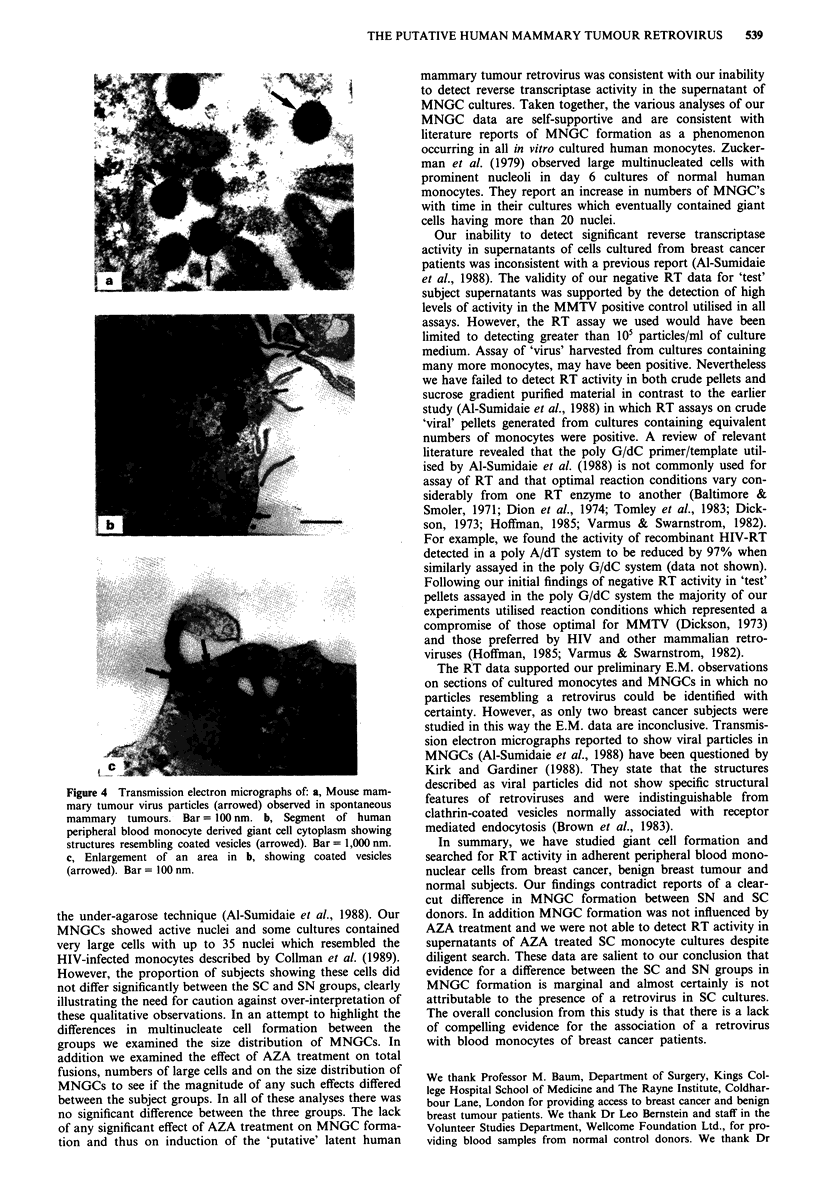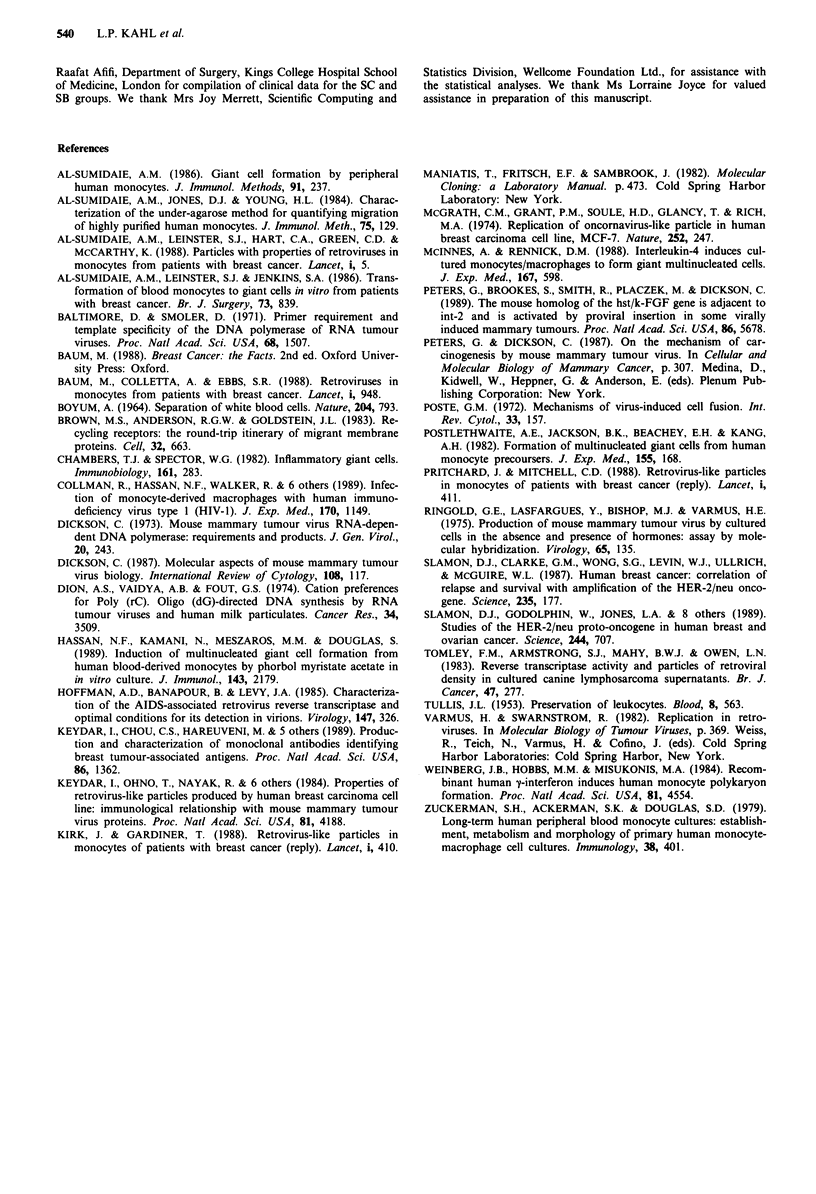# An evaluation of the putative human mammary tumour retrovirus associated with peripheral blood monocytes.

**DOI:** 10.1038/bjc.1991.126

**Published:** 1991-04

**Authors:** L. P. Kahl, A. R. Carroll, P. Rhodes, J. Wood, N. G. Read

**Affiliations:** Department of Virology, Wellcome Research Laboratories, Beckenham, Kent, UK.

## Abstract

**Images:**


Br. J. Cancer (1991), 63, 534–540 © Macmillan Press Ltd., 1991

### An evaluation of the putative human mammary tumour retrovirus associated with peripheral blood monocytes

L.P. Kahl[1], A.R. Carroll[1], P. Rhodes[2], J. Wood[3] & N.G. Read[2]

[1]*Department of Virology,* [2]*Department of Drug Safety Evaluation and* [3]*Department of Scientific Computing and Statistics, Wellcome Research Laboratories, Langley Court, Beckenham, Kent BR3 3BS, UK.*

**Summary** The primary aims of this study were purification and molecular cloning of a putative retrovirus designated human mammary tumour virus (HMTV). However, our preliminary unpublished data of negative reverse transcriptase (RT) activity in ostensibly 'infected' cells led us to re-examine the evidence for this virus; namely multinucleate giant cell (MNGC) formation and RT activity in cultured blood monocytes from breast cancer patients versus benign breast tumour and normal control subjects. MNGCs form by fusion of monocytes and we estimated the total number of cell fusions which had occurred after 10 days of culture *in vitro* by counting cells with two, three, four and five or more nuclei (n) and by measuring the density of adherent mononuclear cells for each subject studied. We found no clear-cut difference in MNGC formation between the three subject groups. Moreover, a substantial number of cultures, encompassing the three groups, showed far more MNGCs per $10^5$ monocytes than previously reported. Various parametric and non-parametric statistical analyses were performed on the multinucleate cell data and only one parametric test, which utilised the density of monolayers as a co-variate, showed a statistically significant difference at the 5% level between the breast cancer and the normal subject groups. We observed marked subject-to-subject variation in multinucleate cell formation and we suggest that the evidence for a difference between the breast cancer and the normal groups is marginal. Further, MNGC formation by breast cancer monocytes may not be attributed to the presence of a retrovirus since 5′-Azacytidine (AZA), an agent known to stimulate replication of latent retroviruses showed no effect on the MNGC formation. In addition, culture supernatants from the three groups were assayed for RT activity and no test sample gave a significant signal above background. Preliminary transmission electron microscopy analysis failed to identify viral particles in MNGCs.

Breast cancer is the commonest cancer in women and in the 35–55 age range it is the principal cause of death amongst women (Baum, 1988). It is currently estimated that 1 in 12 women will develop the disease. Furthermore in the Western hemisphere the annual incidence and mortality rates appear to be on the increase (Baum, 1988).

It has become clear that the clinical onset of breast cancer is influenced by several factors. Amongst these are: (i) the number of menstrual cycles occurring prior to the first full-term pregnancy, (ii) a genetically based predisposition for this disease which is tempered by environmental and cultural factors (Baum, 1988), and (iii) the frequency of spontaneous mutation arising following exposure to ionising radiation (Baum, 1988) and oncogenic transformation (Slamon *et al.*, 1987; Slamon *et al.*, 1989).

One other factor which has represented a major focus of investigation is the potential involvement of a virus of the family Retroviridae in the aetiology of breast cancer. This stems from studies in murine model systems in which a retrovirus designated murine mammary tumour virus (MMTV) has unequivocally been demonstrated to cause breast cancer (see Peters & Dickson, 1987 for review). The virus has been isolated, its entire genome sequenced and much information has been obtained concerning mechanisms by which proviral DNA becomes integrated into the murine genome and the regulation of virus expression (Peters & Dickson, 1987; Dickson, 1987; Peters *et al.*, 1989). The existence of a human mammary tumour virus (HMTV) has been proposed and disputed for many years (McGrath *et al.*, 1974; Al-Sumidaie *et al.*, 1988; Kirk & Gardiner, 1988; Pritchard & Mitchell, 1988; Baum *et al.*, 1988). The positive evidence for this has followed from two lines of investigation. The first involved isolation and characterisation of retrovirus like particles present in the culture fluid of cell lines derived from the pleural effusion of breast cancer patients (McGrath *et al.*, 1974; Keydar *et al.*, 1984; Keydar *et al.*, 1989). A second line of evidence was based on observations of *in vitro* cultured peripheral blood monocytes. Six and 9 day cultures of monocytes from breast cancer patients contained significant numbers of multinucleate giant cells (MNGCs) in comparison with either an absence of, or low numbers in, control cultures (Al-Sumidaie, 1986; Al-Sumidaie *et al.*, 1986). Though notable, these data were not compelling as MNGCs were known to form in tissues due to the presence of a wide variety of infectious agents or the presence of inorganic or organic irritants (Postlethwaite *et al.*, 1982; Poste, 1972; Chambers & Spector, 1982).

Correspondence: L.P. Kahl, Department of Virology, Wellcome Research Laboratories, Langley Court, Beckenham, Kent BR3 3BS, UK.
Received 20 September 1990; and in revised form 28 November 1990.

Our interest in this area was stimulated by the report (Al-Sumidaie *et al.*, 1988) of significant levels of reverse transcriptase (RT) activity in culture supernatants of monocytes from breast cancer patients. This RT activity was associated with particles having a buoyant density similar to that of other retroviruses and was absent from the majority of control cultures. The primary aim of this study was the isolation and molecular cloning of HMTV. However, following our preliminary observations of negative RT activity in cells putatively 'infected' with HMTV, our revised aim was to re-evaluate MNGC formation and RT activity in cultured peripheral blood monocytes from breast cancer, benign breast tumour and normal control subject groups. Our study includes a more comprehensive statistical analysis of multinucleate cell data than that previously described (Al-Sumidaie, 1986; Al-Sumidaie *et al.*, 1986). We report an absence of RT activity and the lack of any clear-cut difference in MNGC formation between the three subject groups.

### Materials and methods

### *Subjects*

The study included 24 female subjects with breast carcinoma (subject cancer, SC), 14 female subjects with current or previous benign breast disease (subject benign, SB) and 24 healthy female volunteer subjects (subject normal, SN) with no evidence of breast disease. Members of the SC and SB groups were of age 27–75 years with 36 subjects being 36 years of age or older. Members of the SN group were of age

34–70 years. Diagnosis of breast cancer or benign breast disease was based on clinical and radiological findings together with a histological examination of the excised tumour.

*Collection of blood*

Thirty millilitres of peripheral venous blood was collected from subjects into three glass vacutaner tubes each containing 10'IU ml$^{-1}$ preservative free heparin. Blood was collected from breast cancer patients prior to surgery and induction of chemotherapy. All blood samples were processed within 2 h of collection, for either MNGC or RT analysis as this volume of blood yielded numbers of mononuclear cells adequate for only one of the two studies.

*Cultivation of adherent blood mononuclear cells*

Mononuclear leucocytes were separated from heparinised blood following its three fold dilution in phosphate buffered (0.01 M) saline (0.15 M) pH 7.2 and centrifugation (800 **g**, 15 min) over a sodium metrizoate (9.6% w/v)/ficoll (5.6% w/v) gradient (Lymphoprep, Nycomed, Torshov, Norway) according to the procedure of Boyum (1964). Mononuclear cells were washed once by centrifugation (400 **g**, 10 min) in tissue culture medium (TCM) composed of Dulbecco's modified Eagles Medium (DMEM, Flow, Rickmansworth, Herts, UK) supplemented with L-glutamine (4 mM), $NaHCO_3$ (3.7 g l$^{-1}$), pencillin (40,000 IU ml$^{-1}$) and streptomycin (20,000 IU ml$^{-1}$). Counts of viable cells were determined by trypan blue exclusion (Tullis, 1953) and cells were adjusted to the appropriate concentration in complete tissue culture medium (CTCM, TCM containing 10% v/v heat inactivated pooled female human AB serum). Cells were plated into Labtek tissue culture slides (Miles Laboratories, Glamorgan, CF31 3TY, UK) as follows: for MNGC analysis cells were brought to $5 \times 10^6$ ml$^{-1}$ and 450 µl dispensed per chamber of an eight chambered tissue culture slide. Two to three chambers were utilised for each time-point studied (days 4 and 10); for RT analysis cells were brought to $7 \times 10^6$ ml$^{-1}$ and 3.5 ml dispensed into each of two one-chambered tissue culture slides. Incubation was at 37°C in an atmosphere of air:$CO_2$ (95:5). After 2 h non-adherent cells were removed by gentle aspiration and the adherent cells washed with warm CTCM thus leaving an enriched monocyte culture. Following a further 12–16 h incubation this washing process was repeated and the medium replaced with appropriate volumes of either CTCM or CTCM supplemented with 15 µM l$^{-1}$ 5' azacytidine (CTCM + AZA) as required (see Figure 1). AZA is known to stimulate the activity of latent retroviruses (Al-Sumidaie *et al.*, 1988). After 7 days of *in vitro* culture the medium was gently aspirated from MNGC cultures and replaced using prewarmed CTCM or CTCM + AZA as indicated in scheme a of Figure 1.

Cultures were fixed following a total of either 4 or 10 days incubation by gentle aspiration of the culture medium which was replaced with prewarmed gluteraldehyde fixative (2% v/v in 0.1 M cacodylate buffer). The adherent cell preparations were stained using May-Grunwald and Giemsa stains (BDH Ltd., Dagenham, Essex, UK) at least 2 h after fixation and then mounted using glass coverslips and DEPEX (BDH). These slides were then used for differential cell counts described below.

For RT analysis adherent mononuclear cells were cultured for 21 days. After 7, 10, 14 and 17 days *in vitro* the culture medium was gently aspirated and replaced with fresh prewarmed CTCM + AZA. Culture supernatants collected at days 7, 10, 14, 17 and 21 were clarified for removal of cellular debris by centrifugation (1250 **g**, 10 mins) and were stored at −70°C prior to RT assay. Figure 1, scheme b illustrates the protocol for maintenance of RT cultures.

*Cell counting and analysis*

Differential counts of the numbers of cell with 2, 3, 4 and 5 or more nuclei in 12 microscope fields (×200) were per-

**Figure 1** Schematic protocol for initiation and maintenance of *in vitro* cultured peripheral blood mononuclear cells for MNGC and RT analysis.

formed on day 10 cultures. We observed that the percentage of adherent cells and thus the monolayer density of day 10 cultures varied between subjects. Multinucleate cells form by fusion of monocytes (Postlethwaite *et al.*, 1982) and the frequency of fusion must be related to the monolayer density. Monocyte density counts were carried out automatically using a SEM-IPS computerised system (Kontron Electrons Ltd, Watford, UK) interfaced to a Zeiss Axiophot photomicroscope (Carl Zeiss (Oberkochen) Ltd., Welwyn Garden City, Herts, AL7 1LU) via a 3CCD colour video camera. Day 10 cultures were not suitable for automated density analysis as by this time very flat and elongated cells predominated and consequently the boundary between individual cells was not always clear. This was particularly the case when large numbers of MNGCs were present. Therefore monocyte density analysis was carried out using the same preparation of each subjects cells following 4 days of *in vitro* culture. This involved an automated count of all monocytes present in 12 fields (×200). The cell analyser was programmed to exclude lymphocytes from cell density counts on the basis of their smaller size and characteristics of nuclear chromatin staining. The 12 microscopic fields for the differential multinucleate cell counts and monocyte density counts were selected such that the same central area of the culture chambers were scored for each subject. The slides were coded and read blind, i.e. without reference to the subject from which the cells had been cultured. The code was necessarily broken after all raw data was collected and prior to the statistical analyses.

*Determination of reverse transcriptase activity*

Crude viral pellets were generated by centrifugation (100,000 **g**, 2 h, 4°C) of pooled culture supernatants which were collected following 7, 10, 14, 17 and 21 days of culture. The crude pellets were taken up in 100 µl of TE (10 mM Tris pH 8.0, 1 mM EDTA) and duplicate 10 µl samples were assayed directly for RT. In later experiments the crude viral pellets were taken up in 500 µl of TE and layered on top of a sucrose step gradient composed of 60% w/v (2 ml), 30% w/v (1 ml) and 15% w/v (1.5 ml). Gradients were centrifuged (85,000 **g**) for 16 h at 4°C. The putative virus was harvested as 2 × 250 µl fractions taken above and below the 30%/60% sucrose interface. The fractions were pooled, diluted and a concentrated viral pellet was generated by centrifugation (150,000 **g**) for 1 h at 4°C. The supernatant was carefully removed and the concentrated viral pellet taken up in TE to approximately 50 µl. RT assays were performed on these preparations.

*Assay for reverse transcriptase*

Following optimisation experiments, reverse transcriptase activity was routinely detected by the incorporation of radiolabelled thymidine or deoxyguanosine triphosphate (TTP or dGTP) into DNA in the presence of a synthetic homopolymer RNA template. Reaction buffer conditions were as follows: 50 mM Tris-HCl, pH 8.3; 50 mM KCl; 8 mM $MgCl_2$; 10 mM DTT; 0.1% NP40; 50µM TTP or dGTP; poly A/dT or poly C/dG at 5 µg $ml^{-1}$; and 10 µCi $^{32}$P-TTP or $^{32}$P-GTP in a final reaction volume of 100 µl. The test sample constituted 50 µl of the final volume. Incubation was for 60 min at 37°C and the reaction was stopped by boiling (30 s). Radiolabelled nucleic acid material was collected by either binding to DE81 discs (Maniatis *et al.*, 1982) or by precipitation using 10% w/v trichloracetic acid. Radioactive incorporation was determined by scintillation counting using a Beckman LS counter Model 1801 (Beckman Instruments, Inc. Palo Alto, CA 94304, USA).

*Mouse mammary tumour virus*

This virus provided a positive control for monitoring yield of virus following the above purification steps and for RT activity in assays. A concentrated preparation of MMTV was kindly provided by Dr Clive Dickson, Imperial Cancer Research Fund, Lincoln's Inn Fields, London WC2A 3PX. It was prepared by collective centrifugation (90,000 *g*, 4°C) of approximately 1200 ml of supernatant from MMTV infected GR cells (Ringold *et al.*, 1975). The crude viral pellet of MMTV was taken up in 1 ml of TE and was stored as 20 µl aliquots at − 70°C. RT assays utilised 10 µl of a 1/100 dilution of this preparation.

*Electron microscopy*

Samples of cultured cells were fixed in 2% glutaraldehyde in 0.1 M cacodylate buffer pH 7.2, were processed for transmission electron microscopy (TEM). Following fixation in glutaraldehyde and buffered 1% osmium tetroxide the cells were washed in cacodylate buffer, dehydrated in acetone and embedded in Spurr epoxy resin in one of two ways. The cells were either scraped off the slides to form a pellet or left as a monolayer and processed *in situ*. Semi-thin (1 µm) sections were cut, stained with methylene blue-azure II-basic fuchsin and examined by light microscopy. Areas of MNGCs were selected in the blocks and thin sections (70–90 nm) were cut, collected on copper/palladium G200 grids, stained with aqueous uranyl acetate and lead citrate (LKB, 2168, Ultrostainer, Cambridge Instruments Ltd., Bar Hill, Cambridge, UK) and examined at an accelerating voltage of 80 KV in a Philips 301 transmission electron microscope.

### Results

*Morphological observations of cultured cells*

Day 10 cultures of the majority of subjects showed cells with two, three, four and ⩾ five nuclei (n) with 2 n and 3 n cells representing a substantial proportion of the multinucleate cell population. Cells typical of those observed at day 10 are shown in Figure 2. Cells with five or more nuclei (⩾5 n) were grouped together when performing differential cell counts, however some subjects showed giant cells with 20–35 nuclei. The three types of MNGCs: foreign body, Langhans and Touton (Chambers & Spector, 1982), were observed in our day 10 cultures with the first two types being seen more frequently than the latter.

In contrast to the deep purple staining of chromatin in monocytes and lymphocytes, MNGCs characteristically showed pale blue staining of their nuclei which invariably contained one to three deep purple nucleoli. Preliminary data (not shown) of cell analysis on day 10 adherent monocyte cultures following silver staining for nucleolar organising regions (NORs) suggest that MNGCs generally contain more (three to five) NORs in comparison with mononucleate cells (one to two NORs, data not shown). Together these morphological observations suggest that the nuclei of MNGCs are active containing DNA which is not supercoiled and in tight association with histone and non-histone proteins.

*Quantitative analyses*

Differential count data from day 10 cultures in CTCM were available from 22 normal, eight benign and 16 cancerous subjects. Of these, 13 normal (SN), five benign (SB) and seven cancerous (SC) also provided day 10 samples in medium supplemented with AZA. There was one additional day 10 SB culture grown in AZA. Usually, there was a day four density reading corresponding to each day 10 culture. However, for two subjects (one SB and one SC with day 10 data in both media), there were no density data available. For six further subjects, there were density readings in only one of the two media for which day 10 data were available. In the following analyses, all the data that are relevant to each question were included. In particular, when adjusting for cell density, a day 4 reading in one medium was used with a day 10 reading in the other if the alternative was to discard data.

The analysis asked three questions:

1. Was there a difference in the rate of formation of MNGCs between the SC, SB and SN groups?
2. Was there a difference in the effect of AZA on the rate of MNGC formation between groups?
3. Was there a difference in the size distribution of multinucleate cells between groups?

*The rate of formation of giant cells* was examined principally by looking at the total number of cell fusion events. This was calculated for each preparation by multiplying the number of cells in a category (two, three, four and five or more nuclei) by the number of fusions that must have occurred to form a cell of that type (in all cases one less than the number of nuclei) and summing these numbers.

i.e. Total fusions = ('2n') + (2x'3n') + (3x'4n') + (4x' ⩾ 5n')

where 'Xn' is the number of cells counted with X nuclei per unit area (= 12 microscopic fields, × 200).

This statistic was biased to some extent because cells with five or more nuclei were all placed in the same category for practical reasons. It could also be argued that only the 'larger' of the multinucleate cells were evidence of the presence of a retrovirus. Therefore the number of 'large' cells (defined as cells with four or more nuclei) observed for each subject was also analysed as in previously reported analyses of similar data (Al-Sumidaie, 1986; Al-Sumidaie *et al.*, 1986).

Both total fusions and number of large cells were analysed (using analysis of variance) on a log scale because the data showed a skewed distribution. Before log-transforming, a small constant (five for fusions and one for large cells) was added to all the observations. The particular values chosen are somewhat arbitrary, but serve to reduce the influence of values close to zero that are disproportionately affected by observational variation.

We observed that the density of glass-adherent cells (which must affect the frequency of cell fusion) varied between subjects, but was not readily estimated from day 10 cultures. Therefore, a subsidiary question was whether the analyses could be improved by adjusting for cell density using the values obtained from the day 4 cultures as a co-variate. Firstly it was necessary to look for differences between the groups in their day 4 density readings, as these would affect the interpretation of any other analyses. Analysis of variance (on a log scale and performed separately for normal and AZA media) revealed no evidence of such differences (data not shown).

**Figure 2** Glass adherent mononuclear and multinucleate cells with two, three, four and ⩾ five nuclei typically seen in day 10 cultures (Magn × 200). Cells with two and three nuclei (arrowed) form a significant component of the multinucleate cell population. **a**, **b**, and **d**. Some cultures contained very large cells with up to 35 nuclei present **c**.

Data revealed a clear association between monolayer density and formation of multinucleate cells (Figure 3). The overall trend was that high monolayer density was associated with large values for total cell fusions and numbers of large cells. This relationship formed a basis for the co-variate analysis performed on total cell fusions and numbers of large cells present in day 10 cultures and the results are shown in Table I. There was an observed trend of increasing values from the normal group through the benign to the cancerous group for both total cell fusions and numbers of large cells (Table I). A direct *t*-test between the SC and SN groups was statistically significant at the 5% level for both analyses, but F-tests were not. Given the large subject to subject variation it might be argued that a larger sample is needed. Nevertheless, the present study does not support the existence of a substantial and clear-cut difference between normal subjects and subjects with malignant disease as reported by others (Al-Sumidaie, 1986; Al-Sumidaie *et al.*, 1986).

Because of some uncertainty surrounding the distribution of the data, equivalent non-parametric tests to the above were performed, but without attempting to adjust for density. These were the Kruskal-Wallis analysis of variance for overall differences between the groups, with Mann-Whitney U-tests for differences between particular pairs. None of these analyses produced a statistically significant result. In addition it was noted that five out of the 16 cancer subjects had cultures containing very large cells with 10 to 35 nuclei. This was higher than the corresponding proportion of the normal subjects (three out of 22), but not significantly different by Fisher's exact test.

**Figure 3** A plot of ln adherent monocyte density *vs* ln total cell fusions which had occurred following *in vitro* cultivation in CTCM for the three subject groups: ● SN; ○ SC; and ▲ SB. A similar plot was obtained for large cell data.

*The effect of 5'-azacytidine (AZA) on multinucleate cell formation* was evaluated using only those subjects for which there was differential cell count data available for both CTCM and CTCM + AZA treatments. The aim of the analysis was to establish whether AZA treatment increased MNGC formation, presumably by promoting viral growth. Further we aimed to determine whether such an effect was

**Table I** Analysis of multinucleate cell formation[a]

| *Total cell fusions* | *SN* | *SB* | *SC* |
|---|---|---|---|
| Mean: log scale | 2.152 | 2.373 | 2.471 |
| s.e: log scale | 0.090 | 0.160 | 0.121 |
| Mean: natural scale | 137 | 231 | 291 |
| *n*:[b] | 22 | 7 | 15 |
| *Numbers of large cells*[c] | *SN* | *SB* | *SC* |
| Mean: log scale | 1.145 | 1.264 | 1.538 |
| s.e.: log scale | 0.123 | 0.217 | 0.149 |
| Mean: natural scale | 13 | 17 | 34 |
| *n*:[b] | 22 | 7 | 15 |

[a]The analysis was based on differential counts of cells cultured for 10 days in CTCM with adjustment for cell density; [b]*n* – number of subjects in group; [c]Large cells have four or more nuclei.

significant in only the cancerous group as this would be consistent with a virus being present in SC cells and absent from SB and SN cells. Both the fusion and large cell data were analysed and the results are shown in Table II. These analyses were not adjusted for density: the comparisons of interest are made within subjects and hence 'automatically' adjusted for density and other between-subject differences. It is clear that there is no evidence of an effect of AZA treatment either overall or differentially between the groups.

*The size distribution of multinucleate cells* between the groups was examined by calculation of the number of cells in each category (two, three, four and five or more nuclei) as a percentage of the total number of multinucleate cells observed for a subject. Analyses of variance for the percent of cells in each category were performed separately for the CTCM and CTCM + AZA treatments. There was no evidence of a difference in the size distribution of multinucleate cells between the SC, SB and SN groups (data not shown).

### *Assay for reverse transcriptase activity*

Supernatants collected from mononuclear cell cultures of a total of 17 subjects were examined for RT activity. Six subjects (three SC, one SB and two SN) were utilised in our earlier RT assays of crude pellet material. Despite the use of both rA/dT and rC/dG primer/template systems no 'test' sample yielded significant incorporation above background. However, an experiment which involved addition of MMTV to 'test' crude pellets revealed an inhibition of MMTV activity. This was attributed to the concentration of non-specific inhibitors from the culture supernatant in the crude pellets. Therefore culture supernatants collected from mononuclear cell cultures of the remaining subjects were submitted to a rA/dT based RT assay following 'virus' purification on sucrose step gradients and concentration of harvested virus. Conditions for this purification were established using MMTV. The remaining subjects totalled 11 (three SC, three SB and five SN) and once again no test sample reproducibly showed significant incorporation above background.

A known quantity of MMTV was run as a positive control throughout virus purification procedures and in all RT assays. Data indicate a 50% yield of virus from the sucrose step gradient. Approximately 50% of this harvested virus was recovered in the final concentrated viral pellet. Calculations based on the RT activity of a known amount of our MMTV positive control together with the sensitivity of its detection in our RT assays indicate that if a human mammary tumour virus was present in our SC cell cultures it must have been at least a 100-fold less active or lower in concentration than the MMTV positive control virus.

**Table II** Analysis of the effect of AZA treatment[a]

| | *CTCM* | *CTCM + AZA* | *n* | *s.e. of the difference* |
|---|---|---|---|---|
| *Total cell fusions* | | | | |
| SN mean: log scale | 2.316 | 2.290 | 13 | 0.070 |
| Mean: natural scale | 202 | 190 | | |
| SB mean: log scale | 2.443 | 2.442 | 5 | 0.112 |
| Mean: natural scale | 272 | 272 | | |
| SC mean: log scale | 2.718 | 2.657 | 7 | 0.095 |
| Mean: natural scale | 517 | 449 | | |
| *Numbers of large cells* | | | | |
| SN mean: log scale | 1.307 | 1.257 | 13 | 0.122 |
| Mean: natural scale | 19 | 17 | | |
| SB mean: log scale | 1.361 | 1.389 | 5 | 0.197 |
| Mean: natural scale | 22 | 24 | | |
| SC mean: log scale | 1.916 | 1.756 | 7 | 0.166 |
| Mean: natural scale | 81 | 56 | | |

[a]Only subjects which provided data in both CTCM and CTCM supplemented with AZA were included in these analyses.

### *Electron microscopy*

Transmission electron microscopy (TEM) was performed on adherent mononuclear and multinucleate cell preparations from two breast cancer patients following 10 or 21 days of *in vitro* culture. TEM of thin sections failed to give conclusive evidence of viral particles in the cytoplasm of the giant cells. No structures consistent with MMTV or other retrovial particles (Figure 4, panel a) were observed. The vesicular structures which were seen resembled the coated vesicles normally present in macrophages and giant cells (Figure 4, panels b and c).

### Discussion

This study, which was initially aimed toward purification and molecular cloning of the described human mammary tumour virus, resulted in an evaluation of MNGC formation and RT activity in cultured blood monocytes and has provided data which differ fundamentally from that previously reported (Al-Sumidaie *et al.*, 1988; Al-Sumidaie, 1986; Al-Sumidaie *et al.*, 1986). We found no clear cut difference in MNGC formation between breast cancer and normal subjects. In contrast we observed significant numbers of MNGCs in virually all cultures from the three groups and many cultures contained far more MNGCs (per $10^5$ adherent monocytes) than previously reported. We found no significant difference in the density per unit area of glass-adherent monolayers between the SC, SB and SN groups. Any significant difference between the groups in their rate of formation of MNGCs is marginal. Direct *t*-tests between SC and SN groups were significant at the 5% level, after adjustment for density, but overall F-tests and non-parametric tests were not. The observed trend of increasing fusions and large cells from the normal group through the benign to the cancerous group is suggestive, but the conclusions from this data set remain equivocal because of the large subject to subject variation. Possibly the greater MNGC formation by SC versus SN monocytes was associated with lymphokine production by T lymphocytes present in the monocyte enriched mononuclear cell preparations studied (Weinberg *et al.*, 1984; McInnes and Rennick, 1988; Hassan *et al.*, 1989).

Cells with two and three nuclei formed a substantial component of the multinucleate cell population in our day 10 cultures and therefore were included in the calculation of total cell fusions. These cells were either not observed or not included in a previous study (Al-Sumidaie *et al.*, 1986) in which MNGCs were defined as having four or more nuclei. However, analysis of our 'large cell' data with omission of 2 n and 3 n cell data did not increase the level of significance between the SC and SN groups.

We used tissue culture slides for cultivation of monocyte enriched cell preparations in preference to the under-agarose cultivation technique described by Al-Sumidaie *et al.* (1984, 1986). Our culture system facilitated development of the three morphological types of MNGCs (Al-Sumidaie, 1986; Chambers & Spector, 1982) and was amenable for use in both morphological and RT analysis. In addition, methods previously described for generation of monocyte culture supernatants for RT analysis clearly did not involve use of

**Figure 4** Transmission electron micrographs of: **a**, Mouse mammary tumour virus particles (arrowed) observed in spontaneous mammary tumours. Bar = 100 nm. **b**, Segment of human peripheral blood monocyte derived giant cell cytoplasm showing structures resembling coated vesicles (arrowed). Bar = 1,000 nm. **c**, Enlargement of an area in **b**, showing coated vesicles (arrowed). Bar = 100 nm.

the under-agarose technique (Al-Sumidaie *et al.*, 1988). Our MNGCs showed active nuclei and some cultures contained very large cells with up to 35 nuclei which resembled the HIV-infected monocytes described by Collman *et al.* (1989). However, the proportion of subjects showing these cells did not differ significantly between the SC and SN groups, clearly illustrating the need for caution against over-interpretation of these qualitative observations. In an attempt to highlight the differences in multinucleate cell formation between the groups we examined the size distribution of MNGCs. In addition we examined the effect of AZA treatment on total fusions, numbers of large cells and on the size distribution of MNGCs to see if the magnitude of any such effects differed between the subject groups. In all of these analyses there was no significant difference between the three groups. The lack of any significant effect of AZA treatment on MNGC formation and thus on induction of the 'putative' latent human mammary tumour retrovirus was consistent with our inability to detect reverse transcriptase activity in the supernatant of MNGC cultures. Taken together, the various analyses of our MNGC data are self-supportive and are consistent with literature reports of MNGC formation as a phenomenon occurring in all *in vitro* cultured human monocytes. Zuckerman *et al.* (1979) observed large multinucleated cells with prominent nucleoli in day 6 cultures of normal human monocytes. They report an increase in numbers of MNGC's with time in their cultures which eventually contained giant cells having more than 20 nuclei.

Our inability to detect significant reverse transcriptase activity in supernatants of cells cultured from breast cancer patients was inconsistent with a previous report (Al-Sumidaie *et al.*, 1988). The validity of our negative RT data for 'test' subject supernatants was supported by the detection of high levels of activity in the MMTV positive control utilised in all assays. However, the RT assay we used would have been limited to detecting greater than $10^5$ particles/ml of culture medium. Assay of 'virus' harvested from cultures containing many more monocytes, may have been positive. Nevertheless we have failed to detect RT activity in both crude pellets and sucrose gradient purified material in contrast to the earlier study (Al-Sumidaie *et al.*, 1988) in which RT assays on crude 'viral' pellets generated from cultures containing equivalent numbers of monocytes were positive. A review of relevant literature revealed that the poly G/dC primer/template utilised by Al-Sumidaie *et al.* (1988) is not commonly used for assay of RT and that optimal reaction conditions vary considerably from one RT enzyme to another (Baltimore & Smoler, 1971; Dion *et al.*, 1974; Tomley *et al.*, 1983; Dickson, 1973; Hoffman, 1985; Varmus & Swarnstrom, 1982). For example, we found the activity of recombinant HIV-RT detected in a poly A/dT system to be reduced by 97% when similarly assayed in the poly G/dC system (data not shown). Following our initial findings of negative RT activity in 'test' pellets assayed in the poly G/dC system the majority of our experiments utilised reaction conditions which represented a compromise of those optimal for MMTV (Dickson, 1973) and those preferred by HIV and other mammalian retroviruses (Hoffman, 1985; Varmus & Swarnstrom, 1982).

The RT data supported our preliminary E.M. observations on sections of cultured monocytes and MNGCs in which no particles resembling a retrovirus could be identified with certainty. However, as only two breast cancer subjects were studied in this way the E.M. data are inconclusive. Transmission electron micrographs reported to show viral particles in MNGCs (Al-Sumidaie *et al.*, 1988) have been questioned by Kirk and Gardiner (1988). They state that the structures described as viral particles did not show specific structural features of retroviruses and were indistinguishable from clathrin-coated vesicles normally associated with receptor mediated endocytosis (Brown *et al.*, 1983).

In summary, we have studied giant cell formation and searched for RT activity in adherent peripheral blood mononuclear cells from breast cancer, benign breast tumour and normal subjects. Our findings contradict reports of a clear-cut difference in MNGC formation between SN and SC donors. In addition MNGC formation was not influenced by AZA treatment and we were not able to detect RT activity in supernatants of AZA treated SC monocyte cultures despite diligent search. These data are salient to our conclusion that evidence for a difference between the SC and SN groups in MNGC formation is marginal and almost certainly is not attributable to the presence of a retrovirus in SC cultures. The overall conclusion from this study is that there is a lack of compelling evidence for the association of a retrovirus with blood monocytes of breast cancer patients.

We thank Professor M. Baum, Department of Surgery, Kings College Hospital School of Medicine and The Rayne Institute, Coldharbour Lane, London for providing access to breast cancer and benign breast tumour patients. We thank Dr Leo Bernstein and staff in the Volunteer Studies Department, Wellcome Foundation Ltd., for providing blood samples from normal control donors. We thank Dr

Raafat Afifi, Department of Surgery, Kings College Hospital School of Medicine, London for compilation of clinical data for the SC and SB groups. We thank Mrs Joy Merrett, Scientific Computing and Statistics Division, Wellcome Foundation Ltd., for assistance with the statistical analyses. We thank Ms Lorraine Joyce for valued assistance in preparation of this manuscript.

### References

AL-SUMIDAIE, A.M. (1986). Giant cell formation by peripheral human monocytes. *J. Immunol. Methods*, **91**, 237.

AL-SUMIDAIE, A.M., JONES, D.J. & YOUNG, H.L. (1984). Characterization of the under-agarose method for quantifying migration of highly purified human monocytes. *J. Immunol. Meth.*, **75**, 129.

AL-SUMIDAIE, A.M., LEINSTER, S.J., HART, C.A., GREEN, C.D. & MCCARTHY, K. (1988). Particles with properties of retroviruses in monocytes from patients with breast cancer. *Lancet*, **i**, 5.

AL-SUMIDAIE, A.M., LEINSTER, S.J. & JENKINS, S.A. (1986). Transformation of blood monocytes to giant cells *in vitro* from patients with breast cancer. *Br. J. Surgery*, **73**, 839.

BALTIMORE, D. & SMOLER, D. (1971). Primer requirement and template specificity of the DNA polymerase of RNA tumour viruses. *Proc. Natl Acad. Sci. USA*, **68**, 1507.

BAUM, M. (1988). *Breast Cancer: the Facts*. 2nd ed. Oxford University Press: Oxford.

BAUM, M., COLLETTA, A. & EBBS, S.R. (1988). Retroviruses in monocytes from patients with breast cancer. *Lancet*, **i**, 948.

BOYUM, A. (1964). Separation of white blood cells. *Nature*, **204**, 793.

BROWN, M.S., ANDERSON, R.G.W. & GOLDSTEIN, J.L. (1983). Recycling receptors: the round-trip itinerary of migrant membrane proteins. *Cell*, **32**, 663.

CHAMBERS, T.J. & SPECTOR, W.G. (1982). Inflammatory giant cells. *Immunobiology*, **161**, 283.

COLLMAN, R., HASSAN, N.F., WALKER, R. & 6 others (1989). Infection of monocyte-derived macrophages with human immunodeficiency virus type 1 (HIV-1). *J. Exp. Med.*, **170**, 1149.

DICKSON, C. (1973). Mouse mammary tumour virus RNA-dependent DNA polymerase: requirements and products. *J. Gen. Virol.*, **20**, 243.

DICKSON, C. (1987). Molecular aspects of mouse mammary tumour virus biology. *International Review of Cytology*, **108**, 117.

DION, A.S., VAIDYA, A.B. & FOUT, G.S. (1974). Cation preferences for Poly (rC). Oligo (dG)-directed DNA synthesis by RNA tumour viruses and human milk particulates. *Cancer Res.*, **34**, 3509.

HASSAN, N.F., KAMANI, N., MESZAROS, M.M. & DOUGLAS, S. (1989). Induction of multinucleated giant cell formation from human blood-derived monocytes by phorbol myristate acetate in *in vitro* culture. *J. Immunol.*, **143**, 2179.

HOFFMAN, A.D., BANAPOUR, B. & LEVY, J.A. (1985). Characterization of the AIDS-associated retrovirus reverse transcriptase and optimal conditions for its detection in virions. *Virology*, **147**, 326.

KEYDAR, I., CHOU, C.S., HAREUVENI, M. & 5 others (1989). Production and characterization of monoclonal antibodies identifying breast tumour-associated antigens. *Proc. Natl Acad. Sci. USA*, **86**, 1362.

KEYDAR, I., OHNO, T., NAYAK, R. & 6 others (1984). Properties of retrovirus-like particles produced by human breast carcinoma cell line: immunological relationship with mouse mammary tumour virus proteins. *Proc. Natl Acad. Sci. USA*, **81**, 4188.

KIRK, J. & GARDINER, T. (1988). Retrovirus-like particles in monocytes of patients with breast cancer (reply). *Lancet*, **i**, 410.

MANIATIS, T., FRITSCH, E.F. & SAMBROOK, J. (1982). *Molecular Cloning: a Laboratory Manual*. p. 473. Cold Spring Harbor Laboratory: New York.

MCGRATH, C.M., GRANT, P.M., SOULE, H.D., GLANCY, T. & RICH, M.A. (1974). Replication of oncornavirus-like particle in human breast carcinoma cell line, MCF-7. *Nature*, **252**, 247.

MCINNES, A. & RENNICK, D.M. (1988). Interleukin-4 induces cultured monocytes/macrophages to form giant multinucleated cells. *J. Exp. Med.*, **167**, 598.

PETERS, G., BROOKES, S., SMITH, R., PLACZEK, M. & DICKSON, C. (1989). The mouse homolog of the hst/k-FGF gene is adjacent to int-2 and is activated by proviral insertion in some virally induced mammary tumours. *Proc. Natl Acad. Sci. USA*, **86**, 5678.

PETERS, G. & DICKSON, C. (1987). On the mechanism of carcinogenesis by mouse mammary tumour virus. In *Cellular and Molecular Biology of Mammary Cancer*, p. 307. Medina, D., Kidwell, W., Heppner, G. & Anderson, E. (eds). Plenum Publishing Corporation: New York.

POSTE, G.M. (1972). Mechanisms of virus-induced cell fusion. *Int. Rev. Cytol.*, **33**, 157.

POSTLETHWAITE, A.E., JACKSON, B.K., BEACHEY, E.H. & KANG, A.H. (1982). Formation of multinucleated giant cells from human monocyte precoursers. *J. Exp. Med.*, **155**, 168.

PRITCHARD, J. & MITCHELL, C.D. (1988). Retrovirus-like particles in monocytes of patients with breast cancer (reply). *Lancet*, **i**, 411.

RINGOLD, G.E., LASFARGUES, Y., BISHOP, M.J. & VARMUS, H.E. (1975). Production of mouse mammary tumour virus by cultured cells in the absence and presence of hormones: assay by molecular hybridization. *Virology*, **65**, 135.

SLAMON, D.J., CLARKE, G.M., WONG, S.G., LEVIN, W.J., ULLRICH, & MCGUIRE, W.L. (1987). Human breast cancer: correlation of relapse and survival with amplification of the HER-2/neu oncogene. *Science*, **235**, 177.

SLAMON, D.J., GODOLPHIN, W., JONES, L.A. & 8 others (1989). Studies of the HER-2/neu proto-oncogene in human breast and ovarian cancer. *Science*, **244**, 707.

TOMLEY, F.M., ARMSTRONG, S.J., MAHY, B.W.J. & OWEN, L.N. (1983). Reverse transcriptase activity and particles of retroviral density in cultured canine lymphosarcoma supernatants. *Br. J. Cancer*, **47**, 277.

TULLIS, J.L. (1953). Preservation of leukocytes. *Blood*, **8**, 563.

VARMUS, H. & SWARNSTROM, R. (1982). Replication in retroviruses. In *Molecular Biology of Tumour Viruses*, p. 369. Weiss, R., Teich, N., Varmus, H. & Cofino, J. (eds). Cold Spring Harbor Laboratories: Cold Spring Harbor, New York.

WEINBERG, J.B., HOBBS, M.M. & MISUKONIS, M.A. (1984). Recombinant human γ-interferon induces human monocyte polykaryon formation. *Proc. Natl Acad. Sci. USA*, **81**, 4554.

ZUCKERMAN, S.H., ACKERMAN, S.K. & DOUGLAS, S.D. (1979). Long-term human peripheral blood monocyte cultures: establishment, metabolism and morphology of primary human monocyte-macrophage cell cultures. *Immunology*, **38**, 401.